# When Less Is More – Discrete Tactile Feedback Dominates Continuous Audio Biofeedback in the Integrated Percept While Controlling a Myoelectric Prosthetic Hand

**DOI:** 10.3389/fnins.2019.00578

**Published:** 2019-06-06

**Authors:** Leonard F. Engels, Ahmed W. Shehata, Erik J. Scheme, Jonathon W. Sensinger, Christian Cipriani

**Affiliations:** ^1^The BioRobotics Institute, Scuola Superiore Sant’Anna, Pisa, Italy; ^2^Division of Physical Medicine and Rehabilitation, Department of Medicine, University of Alberta, Edmonton, AB, Canada; ^3^Department of Electrical and Computer Engineering, Institute of Biomedical Engineering, University of New Brunswick, Fredericton, NB, Canada

**Keywords:** internal model, prosthetics, sensory feedback, psychophysics, motor learning, electromyography

## Abstract

State of the art myoelectric hand prostheses can restore some feedforward motor function to their users, but they cannot yet restore sensory feedback. It has been shown, using psychophysical tests, that multi-modal sensory feedback is readily used in the formation of the users’ representation of the control task in their central nervous system – their internal model. Hence, to fully describe the effect of providing feedback to prosthesis users, not only should functional outcomes be assessed, but so should the internal model. In this study, we compare the complex interactions between two different feedback types, as well as a combination of the two, on the internal model, and the functional performance of naïve participants without limb difference. We show that adding complementary audio biofeedback to visual feedback enables the development of a significantly stronger internal model for controlling a myoelectric hand compared to visual feedback alone, but adding discrete vibrotactile feedback to vision does not. Both types of feedback, however, improved the functional grasping abilities to a similar degree. Contrary to our expectations, when both types of feedback are combined, the discrete vibrotactile feedback seems to dominate the continuous audio feedback. This finding indicates that simply adding sensory information may not necessarily enhance the formation of the internal model in the short term. In fact, it could even degrade it. These results support our argument that assessment of the internal model is crucial to understanding the effects of any type of feedback, although we cannot be sure that the metrics used here describe the internal model exhaustively. Furthermore, all the feedback types tested herein have been proven to provide significant functional benefits to the participants using a myoelectrically controlled robotic hand. This article, therefore, proposes a crucial conceptual and methodological addition to the evaluation of sensory feedback for upper limb prostheses – the internal model – as well as new types of feedback that promise to significantly and considerably improve functional prosthesis control.

## Introduction

The ease with which adults use their hands is owed to an intricate feedforward-feedback mechanism that has been honed since birth (Johansson and Cole, 1992). To those who have lost a hand (i.e., amputees) or were born without it, some feedforward motor functions can be restored with hand prostheses. However, while prostheses with myoelectric control represent the clinical state of the art (Schmidl, 1973), current commercial devices do not intentionally provide sensory feedback, and only few sensory feedback systems have found their way out of the research labs (Antfolk et al., 2013; Clemente et al., 2016; Ortiz-Catalan et al., 2017).

Feedforward control of myoelectric hand prostheses is chiefly influenced by two factors: (1) the robustness of the control of the movements of the prosthesis, which is affected by the method of recording and decoding the users’ intent (i.e., their signals) (Geethanjali, 2016). (2) the users’ ability to produce these control signals that is dependent on their understanding of the system – how it is represented in the central nervous system – which is known as the internal model (Kawato, 1999). In the unimpaired individual, internal models are continuously updated through multi-modal sensory feedback (tactile, visual, and auditory) during and after any movement (Imamizu et al., 2000). In amputees wearing a prosthesis, this differs due to the poor implicit sensory feedback available. Prosthesis users rely, chiefly, on proprioception in the remaining muscles (sense of contraction), visual feedback and, to some extent, on the incidental feedback that motor noise, and socket vibration provide (Simpson, 1973; Childress, 1980; Antfolk et al., 2013; Markovic et al., 2018b). Consequently, they cannot adequately hone their internal model, which negatively affects their ability to control the prosthesis (Lum et al., 2014; Shehata et al., 2018c). When highly reliable efferent signals are available for control, incomplete sensory inputs may suffice to retain the internal model (Hermsdörfer et al., 2008; Saunders and Vijayakumar, 2011; Ninu et al., 2014; Dosen et al., 2015b; Markovic et al., 2018b). However, it is a desirable goal to restore natural closed-loop control with supplementary (explicit) sensory feedback.

To address this goal, researchers have devised and assessed ways of providing feedback through invasive and non-invasive methods (Childress, 1980; Antfolk et al., 2013). Invasive peripheral nerve stimulation holds the promise of eventually being able to restore close-to-natural, modality- and somatotopically matched sensations (Riso, 1999; Graczyk et al., 2016). So far, however, realization of this hope has proven difficult; truly natural “touch” sensations have only been reported once (Tan et al., 2014). Non-invasive feedback does not directly interface with the nerves and is thus potentially less informative, but it is preferred by prospective users (Engdahl et al., 2015). It has also proven capable to improve functional performance in prosthetic hand users (Chatterjee et al., 2008; Ninu et al., 2014; Raspopovic et al., 2014; Clemente et al., 2016; Dosen et al., 2016; Markovic et al., 2017, 2018a). All these studies demonstrated new technological devices and methods, produced new knowledge, and revived the interesting question on the need/effectiveness of sensory feedback and how to assess it. However, no study had assessed the effects of sensory feedback on the internal model within a formalized framework.

In an attempt to reduce this gap, we recently proposed to assess the internal model strength developed while controlling myoelectric prostheses by using a psychophysical framework borrowed and modified from motor adaptation studies (Johnson et al., 2017; Shehata et al., 2018a,c). This framework uses parameters, such as sensory and control noise, to compute uncertainties in the developed internal model. Our recent work (Shehata et al., 2018c) showed that this framework can be used to investigate the effect of the feedback level on internal model strength. As a test bed for assessing this new method, we developed a versatile non-invasive human-machine interface that included a classifier for control and an audio sensory feedback system conveying continuous information about the control inputs of the classifier (EMG biofeedback) (Shehata et al., 2018a,b). The psychophysical framework proved that the strength of the internal model depends on the sensory input received (Shehata et al., 2018c). In particular, it showed that when audio biofeedback was added to vision, it outperformed the visual feedback alone in terms of internal model strength and performance in a functional task – both in a virtual environment and while using a multi-DoF hand prosthesis (Shehata et al., 2018a,b).

Based on these results, we sought to further enhance the sensory input available to the user, with complementary cues, in order to assess whether and how this could result in an even stronger internal model, and better performance in a functional task. To this aim we assessed and compared four sensory feedback conditions while controlling a myoelectric research hand prosthesis in psychophysical and functional tests. The three main conditions differed regarding the amount of complementary information: “visual-only (V),” “visual-plus-audio (VA),” and “visual-plus-audio-plus-tactile (VAT).” To disentangle the effects of the tactile component on the outcomes of the VAT feedback, the fourth condition was “visual-plus-tactile (VT).” The tactile feedback was provided by means of short-lasting vibrotactile cues (time-discrete) rather than continuous feedback, according to our previous work (Cipriani et al., 2014; Crea et al., 2015; Clemente et al., 2016; Barone et al., 2017; Aboseria et al., 2018) and the *discrete event-driven sensory feedback control* (DESC) policy (Johansson and Cole, 1992; Johansson and Edin, 1993; Johansson and Flanagan, 2009). The latter is a neuroscientific hypothesis of the mechanisms involved in human sensorimotor control, which posits that manipulation tasks are organized by means of multi-modally encoded discrete sensory events, e.g., resulting from object contact and lift-off.

Our findings show that all augmented feedback types significantly improved the performance compared to vision alone in the functional task, but only the audio biofeedback (VA) had an effect on the internal model strength, as measured by the psychophysical framework/metrics. Conversely, the tactile feedback demonstrated poor psychophysical metrics without (VT) and in combination with the audio biofeedback (VAT). These results on how the different inputs combine (either constructively or destructively) in the integrated sensory percept contribute to the scientific debate on the internal model and suggest ways for providing effective supplementary sensory feedback to prosthetic hand users.

## Materials and Methods

### Study Participants

We collected data from 28 healthy participants without any limb difference [13 females; age: 25 ± 4.5 (mean and standard deviation)]. All participants had normal or corrected-to-normal vision, were right-handed, and had no previous experience with myoelectric control. We had already collected the data for the “visual-only (V)” and “visual-plus-audio (VA)” groups (14 participants) and presented some aspects of it in our previous study (Shehata et al., 2018a). Written informed consent according to the University of New Brunswick Research and Ethics Board and the Scuola Superiore Sant’Anna Ethical Committee was obtained from all participants before conducting the experiments (UNB REB 2014-019 and SSSA 02/2017). The protocol used in this study was approved by the University of New Brunswick Research and Ethics Board and the Scuola Superiore Sant’Anna Ethical Committee.

### Experimental Setup

**FIGURE 1:**
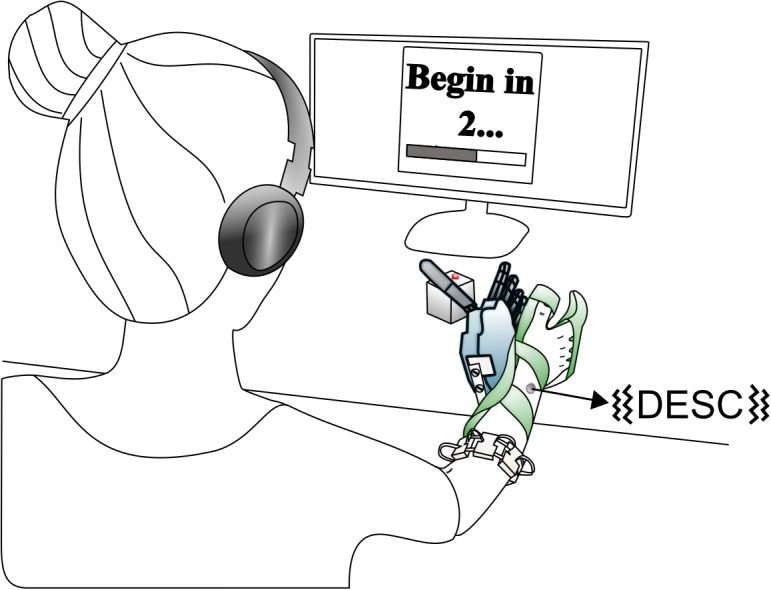
Overview of the setup. The robotic hand was attached to the participant via bypass. The EMG signals recorded from the electrode bracelet around the forearm controlled the hand. A vibrotactor on the dorsal forearm provided discrete feedback i.e., discrete event-driven sensory feedback control (DESC), and the headphones provided continuous feedback. If the grasping force on the test object exceeded a breaking threshold in fragile mode, its red LED turned on. Modified from Shehata et al. (2018a), used under CC BY 4.0.

The experimental setup was similar to that of our previous study (Shehata et al., 2018a) and is briefly described here. It comprised an array of eight custom-made myoelectric sensors in a bracelet; a right-handed sensorized research hand prosthesis (IH2 Azzurra hand, Prensilia S.r.l., IT) that was mounted on a bypass attached to the participant’s forearm; a PC running the control and feedback algorithms; standard commercial headphones (MDRZX100, Sony, JP) for the audio feedback; a vibrotactor for the tactile feedback (Pico Vibe 312-101, Precision Microdrives, United Kingdom); and an instrumented test object [57 mm × 57 mm × 57 mm, ca. 180 g; (Controzzi et al., 2017; Figure 1)].

**FIGURE 2:**
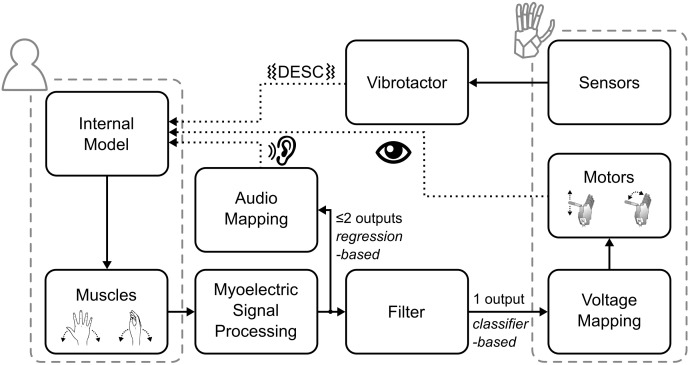
Overview of the control and feedback loop. The dotted lines indicate the three types of feedback provided to the participants.

The myoelectric sensor bracelet was placed around the forearm of the participant and recorded the muscle activity used to control the robotic hand. We limited robotic hand movements to two degrees of freedom (DoF): (1) flexion/extension of the thumb, index and middle fingers, and (2) abduction/adduction of the thumb. Each of the four directions of movement of the robotic hand was mapped to one of four specific wrist movements: flexion and extension of the wrist corresponded to flexion/extension of the digits, while wrist abduction and adduction corresponded to abduction/adduction of the thumb. To implement the mapping, i.e., to interpret the electromyographic (EMG) signals, we used a Support Vector Regression algorithm that provided two regression-based control signals, which could simultaneously activate the two DoFs (e.g., thumb adduction and finger flexion) (Shehata et al., 2017, 2018c). These signals were then gated by a classifier; that means, the hand only moved in one direction at a time (Figure 2).

Biofeedback is the technique of providing biological information to participants in real-time that would otherwise be unknown (Giggins et al., 2013). Accordingly, the audio biofeedback continuously relayed the two outputs of the regression-based controller to the participant, in the form of four distinct tones. Wrist flexion and extension were mapped to tones of 400 and 500 Hz, wrist abduction and adduction to 800 and 900 Hz, respectively. The amplitude of the tones was proportional to the output of the regression algorithm (max volume = 53 ± 3 dB Sound Pressure Level). With this architecture (Figure 2), while the hand moved only one DoF at a time, the audio biofeedback provided richer information about the participant’s myoelectric signals, which encompassed both proprioceptive and motor output information.

The tactile feedback provided information about the physical interactions of the robotic hand with the environment through the vibrotactor on the dorsal forearm. It delivered a short-lasting vibration burst (60 ms, 150 Hz, peak-to-peak force amplitude of ca. 0.3 N) upon contact, liftoff, replacement, and release of the test object. These events are known to be highly significant for the normal grasp-and-lift control, as per the DESC policy (Johansson and Cole, 1992; Johansson and Edin, 1993; Cipriani et al., 2014).

The test object – called an iVE – contained load cells measuring the grasp and load forces. The iVE could virtually break when a grasp exceeded a force of ca. 3 N, which was signaled to the participant by the activation of a red-colored LED on the iVE (Controzzi et al., 2017).

### Experimental Protocol

Participants were divided into four groups (7 persons each) according to the kind of feedback they received: “visual-only (V),” “visual-plus-audio (VA),” “visual-plus-tactile (VT),” and “visual-plus-tactile-plus-audio (VAT).” They performed two tests according to a previously developed psychophysical framework (Shehata et al., 2018c): the “adaptation rate test” to measure the rate of optimization of grasping due to the feedback, and the “just-noticeable difference (JND) test” to measure the threshold of perceiving a control perturbation. A third “functional test” was added in order to measure the ability to use the robotic hand in a manipulation task (Clemente et al., 2016).

In the adaptation rate test, the participants had 40 trials and were asked to grasp, lift, and replace the iVE as quickly as possible without breaking it. The iVE was placed in front of the participant so that the LED faced upward and the two instrumented sides could be grasped without the need to turn the object. A limit of 5 s was given to execute each trial, after which the hand automatically reopened. During trials 1–25, breaking was signaled through a red LED (fragile mode); during trials 26–40, the breaking was no longer signaled (rigid mode). This was done to keep subjects engaged with the task and prepare them for the following test.

In the JND test, the participants grasped the iVE (fragile mode) in two consecutive trials, lasting 4 s each (after which the hand automatically reopened). In one of the two, a stimulus perturbed the control of the hand. Participants were told to identify the altered trial (two-alternative forced choice) by pressing a key on a keypad placed near their unconstrained hand. The stimulus was calculated using an adaptive staircase procedure with a target probability set to 0.84 and a step size of 67 degrees and repeated until 23 reversals were achieved, as in our previous work (Shehata et al., 2018c).

In the functional test, for 20 trials of 10 s each, the participants attempted to grasp, and transfer the iVE over a barrier (H: 14.5 cm × W: 25 cm) without breaking it (fragile mode), akin to the well-known Box and Block test (Mathiowetz et al., 1985; Clemente et al., 2016). For a more detailed description of the tests please refer to (Shehata et al., 2018a).

In all groups, participants first trained freely to become familiar with the control and then trained to grasp and lift the test object. After that, they completed the three tests receiving only visual feedback. Subsequently, participants repeated the training and the three tests with either V, VA, VT, or VAT feedback. Ergo, each participant completed training and the three tests twice. The four groups were thus different and received the following feedback (in order): V-V, V-VA, V-VT, and V-VAT. In between the tests, the participants took short breaks; they took additional breaks during the (long-lasting) JND (every 12 min, or more often if desired). Each trial of the three tests was started with the hand fully opened and ended with the hand (automatically) returning to this starting pose. In the adaptation rate test and in the JND test, the thumb was fully adducted, meaning that participants had to activate only one DoF to close the hand [see also **Figure 3** in Shehata et al., 2018a]. In the functional test, the fingers were extended, and the thumb fully abducted in resting position, meaning that the participants had to activate the two DoFs, mimicking the control of a multi-DoF prosthetic hand.

**FIGURE 3:**
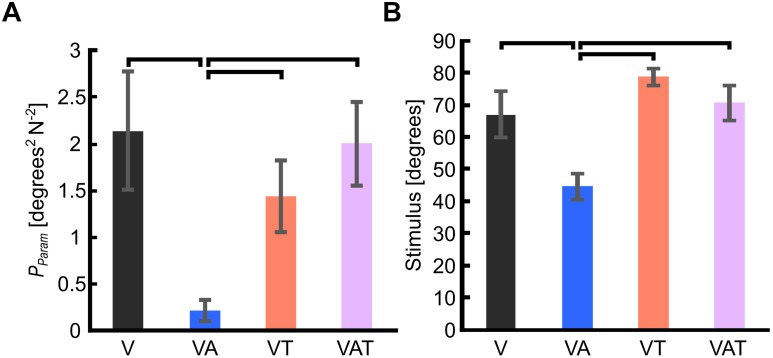
**(A)** Internal model uncertainty (*P*_param_). **(B)** Sensorimotor threshold (JND). Horizontal bars denote *p* < 0.05. Error bars show the standard error of the mean for each group.

### Outcome Measures

The internal model developed while using the robotic hand was reconstructed from the data of the second repetition of the tests, by extracting four metrics from the adaptation rate and the JND tests, following the procedure described in our previous study (Shehata et al., 2018a). These are termed *psychophysical metrics* and consist of:

• *P*_param_: Internal model uncertainty. This measure describes the confidence participants have in their developed internal model, and it is computed from the outcomes of the adaptation rate test and the JND test (Shehata et al., 2018c and their Supplementary Materials).
• JND: Just-noticeable difference (or sensorimotor threshold). It describes, in degrees, the smallest external control perturbation from the trajectory (generated by the participant) that the participant perceived. The JND was defined as the final noticeable stimulus after 23 reversals of the adaptive staircase in the JND test (Shehata et al., 2018c).
• R: Sensory uncertainty. R determines the participants’ trust in the sensory information they receive from the system (Shehata et al., 2018c). It is derived from the JND and the controller noise (Q) as follows:
  (1) $$R=\frac{{\mathrm{JND}}^{2}}{2}-Q$$

Q was extracted from the adaptation rate test as the variance in the control signal between the start of each trial and the first activation of the muscles (ca. 100–200 ms).

• −*β*_1_: Adaptation rate. This is a measure of the participants’ modification of the feedforward control signal (from one trial to the next) based on the perceived error between the optimal and their actual movement (Bastian, 2008; Johnson et al., 2017). It was computed from each trial in the adaptation rate test by analyzing the first 150–300 ms window of the output signal from the controller (Shehata et al., 2018b). This window was selected to truly assess modifications in the control signals before integration of the visual feedback (Elliott and Allard, 1985). Since this test required only flexion of the digits, any other activations were considered self-generated errors (Shehata et al., 2017). Participants were incentivized to minimize these errors while executing the task as quick as possible without (virtually) breaking the object. We computed the −*β*_1_ as follows:
  (2) $${\mathrm{error}}_{n+1}-{\mathrm{error}}_{n}=\beta_{1}\times {\mathrm{error}}_{n}+\beta_{0}$$

where error is the angle between the ideal and the actual hand trajectory, *β*_0_ is the linear regression constant, *β*_1_ is the adaptation rate, and n is the trial number. A unity value for *β*_1_ indicates perfect adaptation, i.e., an internal model that is modified to perfectly compensate for errors. Higher or lower adaptation rates suggest over- or under-compensation.

In addition – to infer the way participants used the sensory input to control the robotic hand to grasp and lift the object – we computed the number of sub-movements from the second block of data in the adaptation test. Sub-movements are defined as the number of zero-crossing pairs of the third derivative of the grasp force profile per trial (Fishbach et al., 2007). This measure describes the real-time (or closed-loop) regulation of the grasp force and depends on the received feedback (Doeringer and Hogan, 1998; Kositsky and Barto, 2001; Dipietro et al., 2009). Specifically, a higher number of sub-movements indicates closed-loop regulation of the grasp force.

Finally, the completion rate (defined as the proportion of successful transfers) and the mean completion time (the average time needed to successfully transfer the object) were computed from the second repetition of the functional test, akin to our previous studies (Clemente et al., 2016).

### Statistical Analysis

We tested all parameters for homogeneity in variances of the data by using Levene’s test in SPSS (IBM Corp., United States). If data variances were homogenous, one-way ANOVAs were used to assess differences among metrics for the feedback types tested. If statistical significance was found, Bonferroni *post hoc* analysis test was performed. However, if data variances were found to be non-homogeneous, robust Welch ANOVA was used instead and followed by *post hoc* analysis using Games-Howell test. The confidence interval was calculated using the standard deviation (95% CI = mean ± 1.96 × SD).

## Results

The internal model uncertainty, *P*_param_, proved significantly lower with the audio augmented feedback (VA) compared to all other conditions [robust Welch ANOVA (F (3, 24) = 8.6, *p* = 0.006) and Games-Howell *post hoc* tests *p* < 0.05] (Figure 3A). No other statistical differences were observed. In contrast to our expectations, with VAT, *P*_param_ (2.0 ± 0.45) was larger than VA (0.22 ± 0.11) and VT (1.4 ± 0.4), and not statistically different from V (2.14 ± 0.64). In other words, adding the tactile component to the audio biofeedback not only produced a lower confidence on the internal model than the two components (VA and VT) individually, but it degraded to the level of visual feedback alone.

The JND was 67 ± 7.2 degrees for V, 44.6 ± 3.9 degrees for VA, 78.6 ± 2.5 degrees for VT, and 70.6 ± 5.4 degrees for VAT. Its trend matched with that of *P*_param_. Indeed, it was lowest for the VA condition (one-way ANOVA with Bonferroni *post hoc* tests, *p* < 0.05), while no other statistical differences were found among the conditions (Figure 3B).

**FIGURE 4:**
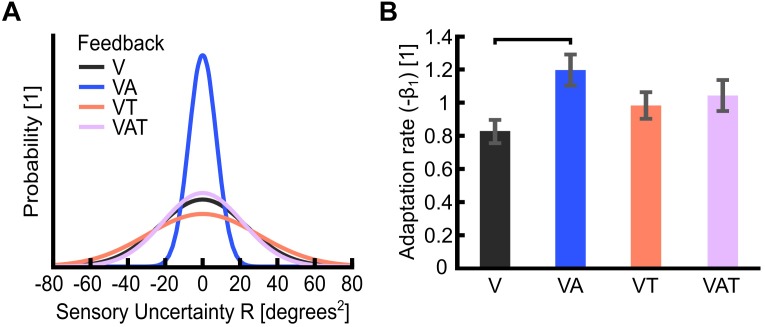
**(A)** Sensory uncertainty, R. The graph is a visualization of the variance of each feedback strategy in the probability curve. It displays Gaussian curves constructed with a variance of R and an arbitrary area under the curve of 1 unit. Visual-plus-audio biofeedback (VA) shows the narrowest variance, i.e., the lowest uncertainty. Conversely, visual-plus-tactile (VT) shows the widest variance, i.e., the highest uncertainty. **(B)** Adaptation rate (−*β*_1_). The adaptation rate describes the extent to which participants adapted to the self-generated error, i.e., how well they could optimize their control from one trial to the next. Horizontal bars denote *p* < 0.05. Error bars show the standard error of the mean for each group.

Akin to *P*_param_ and JND, the sensory uncertainty (R) was lowest under VA and largest under VT (Figure 4A).

The adaptation rate was 0.82 ± 0.1 for V, 1.2 ± 0.1 for VA, 0.98 ± 0.1 for VT and 1.03 ± 0.1 for VAT. These outcomes indicated that, when using the VA feedback, participants adapted more than when using the other types, although this difference was statistically significant only in comparison to V (Figure 4B; Shehata et al., 2018a). No other statistical differences could be observed across conditions. It is worth noting that the controller noise Q extracted from this test was consistent across all conditions (<20%; not shown). This means that any variability in R is an effect of the sensory feedback, not of the variability of Q.

Altogether, the psychophysical metrics all indicated VA as the condition yielding the strongest internal model, as assessed by a lower JND and sensory uncertainties, higher adaptation rate, and a stronger internal model.

**FIGURE 5:**
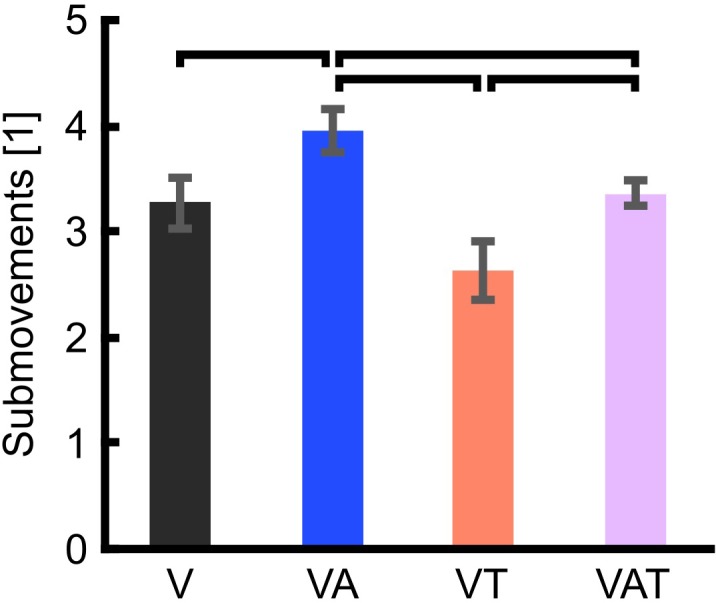
Trial sub-movements. The graph shows significantly more sub-movements with VA than with all other feedback types. VAT lead to significantly more sub-movements than VT. VT and V are not statistically different. Horizontal bars denote *p* < 0.05. Error bars show the standard error of the mean for each group.

**FIGURE 6:**
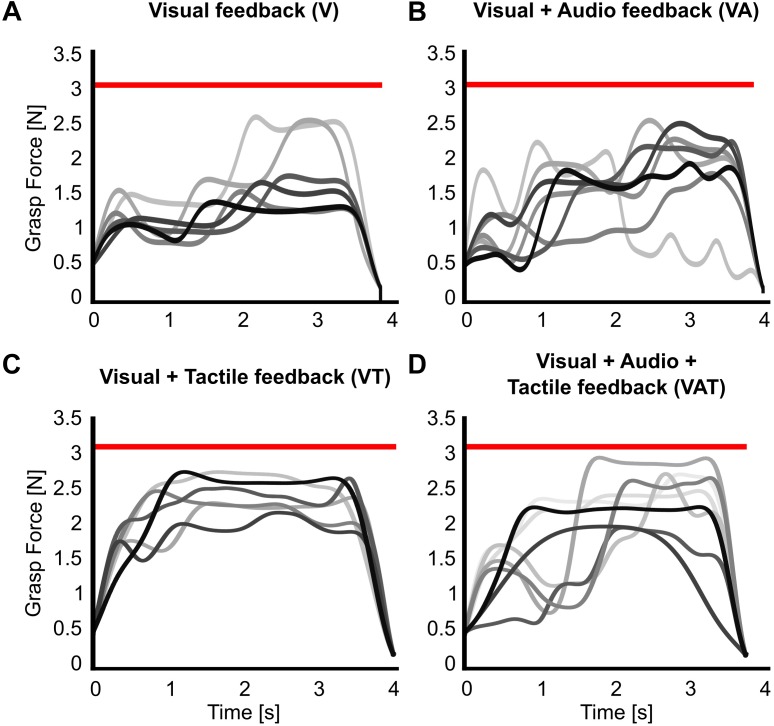
Grasp force profiles. Representative grasp force profiles from individual trials during the adaptation rate test for all feedback types. A participant using **(A)** only visual feedback had lower variance in their grasping patterns over trials, **(B)** visual-plus-audio adjusted their grasping force throughout the trials and had higher variance in their grasping patterns, **(C)** visual-plus-tactile had automated grasping patterns with very low variance, and **(D)** visual-plus-audio-plus-tactile seemed to have highly variable grasping pattern in the initial trials but more automated grasping pattern at the final trials. Earlier trials are in lighter, later trials in darker shades of gray. The red horizontal bar indicates the breaking threshold. Incomplete trials in which the iVE was broken are not shown.

The analysis on the sub-movements highlighted a statistically significant difference across conditions (one-way ANOVA; *p* = 0.001) (Figure 5). Participants using VA adjusted their control signals significantly more than participants using the other feedback types (Bonferroni *post hoc* tests, *p* < 0.05). In other words, the participants tended to use the audio biofeedback in real-time, in order to modulate their grasp force to prevent breaking the object. Conversely, participants using the VT performed significantly less sub-movements than participants using the audio biofeedback (VAT and VA) (Bonferroni *post hoc* tests, *p* < 0.05). No other statistical differences were observed. These general behaviors were nicely captured by the time series of the grasp forces during the adaptation rate test (Figure 6). The evolution of the grasp force profiles, under the different conditions, suggest that, in the VAT, the participants used the audio biofeedback in the initial trials (light gray traces in Figure 6), and the discrete tactile feedback in the later trials (dark gray traces).

**FIGURE 7:**
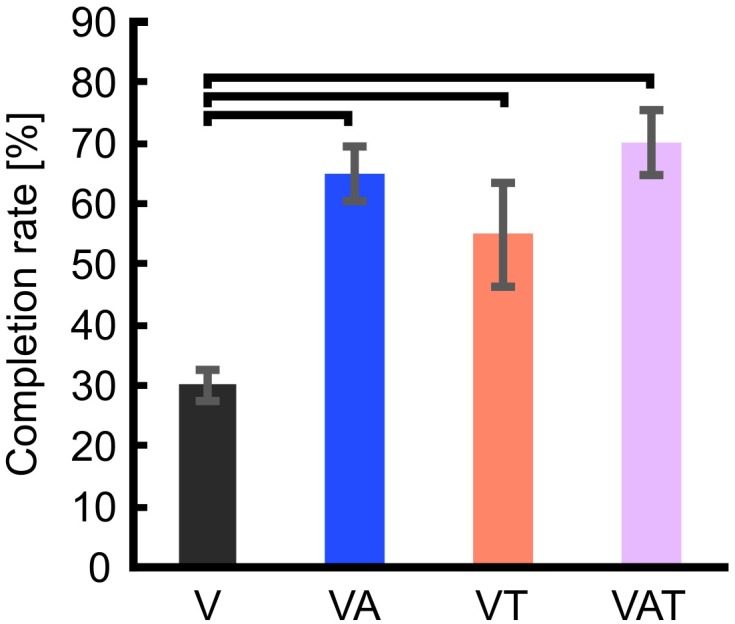
Completion rate. The figure shows that the percentage of successful transfers (i.e., the iVE was not broken) was significantly higher when the participants received any kind of augmented feedback compared to visual alone. Horizontal bars denote *p* < 0.05. Error bars show the standard error of the mean for each group.

Regarding the functional test, the completion rate with visual feedback only (V) proved significanly worse than with VA, VT, and VAT (One-way ANOVA; Bonferroni *post hoc* tests, *p* < 0.05). Further, there may be a slight trend toward more successful transfers with VAT (70 ± 5.4) compared to VT (55 ± 8.5) and VA (65 ± 4.6) but it did not reach significance (*p* = 0.3 and *p* = 0.6, respectively) (Figure 7).

The mean completion time was not affected by the different feedback types and was 8.4 ± 0.65 s for V, 8.3 ± 0.74 s for VA, 8.6 ± 0.32 s for VT, and 8.4 ± 0.34 s for VAT.

## Discussion

Many researchers have explored ways of providing hand prostheses with supplementary sensory feedback, showing that it could indeed improve the performance in functional tasks. Yet, very little consideration has been given to the causes underpinning improved performance; in particular, it is still unknown how feedback contributes and combines to build strong internal models of the myoelectric control system. Here, we hypothesized that increasing the sensory information provided to a myoelectric hand user could result in a stronger internal model and better performance in a functional task. Hence, we combined continuous audio biofeedback with event-based vibratory tactile feedback in a myocontrolled prosthetic hand. Furthermore, we also explored the complex interactions between different feedback types (i.e., continuous visual and audio, and discrete tactile feedback) and their effects on the internal model strength.

Audio biofeedback provided continuous information about intensity of the control signal but not about the actual grasp, whereas time-discrete tactile feedback exclusively conveyed precise information about the interactions between the robotic hand and the environment. According to Johansson and colleagues (reviewed in Johansson and Flanagan, 2009), these interactions are processed and signaled to the nervous system through discrete sensory events and are crucial for developing efficient and natural feed-forward grasping in humans.

In this study we confirmed our previous findings (Shehata et al., 2018a): adding complementary audio biofeedback to visual feedback enables the development of a stronger internal model for controlling a myoelectric hand, as assessed by all psychophysical metrics (*P*_param_, JND, R, and −*β*_1_). The fact that the VA feedback yielded a lower sensory uncertainty (or variance) than V (cf. Figure 3A) suggests that the audio component dominates the integrated visual-audio percept, according to the maximum-likelihood estimate theory (Ernst and Banks, 2002). In other words, when the visual input is complemented with a coherent audio biofeedback, participants would likely rely more on the latter to execute the motor task. This is in agreement with current understanding of human sensorimotor control: the nervous system can never be completely certain about the relevance of visual information, as it provides only indirect information about the motor task, and the interactions with the environment (Johansson and Flanagan, 2009; Wei and Kording, 2009). Whether these results are due to the modality of the biofeedback (i.e., audio) or to the nature of the biofeedback itself (i.e., the sensory input which closely matches the intended motor output), remains to be assessed. Interestingly our results align with those of Dosen et al. (2015a), who conveyed EMG biofeedback using a visual interface.

The reconstructed internal model did not further improve when another piece of redundant information – this time about the touch event – was added to visual and audio biofeedback. In fact, the VAT condition yielded significantly worse psychophysical metrics compared to VA, showing results closer to the basic condition V (and also to VT). These results – if the psychophysical metrics are a truthful and complete description of the internal model – indicate that adding sensory information, albeit consistent with the already available information, may not necessarily enhance the formation of the internal model in the short term. In fact, it could even degrade it. A possible explanation for this is given by the causal inference hypothesis (Knill, 2003; Ernst, 2006; Körding et al., 2007), which posits that the nervous system interprets cues in terms of their causes. When the cues are very different from one another in space and time, the nervous system will infer that they are not related and thus should be processed separately. The visual and audio cues were indeed caused by the same process (i.e., the control input) while the tactile cues were due to the interaction of the robot hand with the environment (the control input is also causal of touch but through a transformation that involves extrinsic factors as well).

Combined in the VAT, the tactile component apparently dominated in the so-integrated percept, as indicated by the sub-movements and grasp force profiles (Figure 5, 6) and also shown by the clear degradation of the psychophysical metrics. This suggests that, when combined and during grasping, (extrinsic) tactile sensory cues are more relevant to the central nervous system than (intrinsic) biofeedback cues – at least in the time frame explored. It is interesting to observe that this degradation was not immediate, as the tactile feedback only became predominant after several trials (Figure 6). This could mean that, when both types are present, audio biofeedback may be easier to pick-up in the initial phases – perhaps because it is very informative and closely matches the motor output – whereas it becomes less relevant in the later stages – possibly because it is more cognitively taxing compared to the tactile input. This argument would be supported by the literature on motor adaptation (Wei and Kording, 2009) and sensorimotor control of dexterous manipulation tasks (Johansson and Flanagan, 2009). Another possible reason for favoring the continuous feedback in the initial phases is related to how people expect to receive information of the grasp based on their top-down knowledge of the interactions of the body with the environment: in nature these interactions are continuous (although they may be processed differently by the nervous system). However, why and how the tactile input corrupted the psychophysical metrics (instead of enhancing them), remains unclear so far. Future tests could investigate whether the internal model is updated more efficiently with audio biofeedback than with tactile feedback after disturbances, for example by doing a pick-and-lift task with unexpectedly changing object weight (Jenmalm et al., 2006).

The degradation of the psychophysical metrics with VAT is, nevertheless, interesting, as one would expect that the tactile feedback should barely interfere in such tests, contrary to what we observed. The JND tested how well the participants could perceive discrepancies in the control input. Here, audio biofeedback provided a lot of relevant information, visual some (because the hand was not always, and completely under visual control) but tactile only notified the participants about touching the object (which was expected to be meaningless in the JND). In the adaptation rate test – where the task was to grasp and lift the object – tactile info conveyed some more information about the final result of the control, i.e., a successful or unsuccessful grasp. In fact, in the adaptation rate test, tactile information (VT and VAT) yielded optimal results (−*β*_1_ close to 1) whereas with only audio feedback (VA) participants overcompensated (−*β*_1_ > 1). While the near-perfect adaptation with VT and VAT may be due to the saliency of the time-discrete sensory feedback policy (Johansson and Cole, 1992; Johansson and Edin, 1993; Johansson and Flanagan, 2009), we argue that audio feedback alone – being a reliable and continuous sensory input coherent with vision – induced the participants to adapt continuously. However, it is still unclear whether this difference between VA and VT/VAT (and V and VT/VAT) is meaningful.

The results from the functional test were complementary to those from the psychophysical tests. We found that all kinds of augmented feedback (VA, VAT, and VT) enabled users to perform significantly better than with vision alone (Figure 7). It was expected that VT would allow for better performance than V alone (Clemente et al., 2016). Further, this result advocates that continuous audio biofeedback can enhance motor control of a myoelectric prosthesis [in agreement with the work of Dosen et al. (2015a) and our previous study (Shehata et al., 2018a)]. However, it also reveals a significant deviation from the results of the other tests. Indeed, while participants with VT or VAT integrated the sensory input and exhibited a predictive control behavior, participants with VA used it for continuously regulating their grip force in real-time, in a closed-loop manner (as seen in the data from the adaptation test in Figure 5, 6B). We believe this was due to the nature of the feedback: the time-discrete sensory cues could only be used by participants as checkpoints for the motor task (Johansson and Edin, 1993; Clemente et al., 2016), whereas the audio biofeedback – as discussed above – induced the participants to use it constantly, even when the grasp was successful. Both approaches seem to be equally adequate to improve grasping performance with a prosthetic hand.

During object manipulation, the brain uses sensory predictions and afferent signals to adapt the motor output to the physical properties of the manipulated object, as well as to monitor and update task performance (Johansson and Flanagan, 2009). In this way, humans can predict and use an adequate level of grip force required to lift an object by producing highly coordinated grasping and lifting forces and correcting their actions in the case of unexpected events (e.g., object slip or incorrectly predicted weight). Sensory feedback plays a crucial role in building and keeping the motor control repertoire updated. However, neural delays make continuous closed-loop control of dynamic motor behaviors impractical at frequencies above 1 Hz (Hogan et al., 1987; Johansson and Edin, 1993). Hence, natural grasping largely involves predictive feedforward rather than closed-loop (servo control) mechanisms. With this in mind, and considering that the VAT and VT yielded a successful predictive control behavior in the functional test (a sign of a mature internal model), we suspect that the psychophysical tests used may not grasp all the facets of the internal model. In particular, they may not be capable to properly assess the contribution of touch-related sensory information, or, alternatively, the discrete tactile feedback may have masked the measurements.

## Ethics Statement

Written informed consent according to the University of New Brunswick Research and Ethics Board and the Scuola Superiore Sant’Anna Ethical Committee was obtained from all participants before conducting the experiments (UNB REB 2014-019 and SSSA 02/2017). This work is not a clinical trial. All experiments were conducted in a lab and not in a clinic.

## Author Contributions

All authors took part in conceiving and designing the study, discussed the results, and contributed to manuscript revision, read, and approved the submitted version. AS and LE conducted the study and wrote the manuscript. AS analyzed the data.

## Conflict of Interest Statement

CC is co-founder and holds shares of Prensilia S.r.l., which manufactures and sells the IH2 Azzurra hand used in this study. The remaining authors declare that the research was conducted in the absence of any commercial or financial relationships that could be construed as a potential conflict of interest.

## Abbreviations

DESC: discrete event-driven sensory control
DoF: degree of freedom
EMG: electromyography/electromyographic
iVE: instrumented virtual egg
V: visual feedback
VA: visual + audio biofeedback
VAT: visual + audio + tactile feedback
VT: visual + tactile feedback.
